# Electroconvulsive therapy (ECT) for the treatment of fibromyalgia (FM): a prospective randomized single-blind, sham-controlled study

**DOI:** 10.1192/j.eurpsy.2026.10932

**Published:** 2026-07-31

**Authors:** E. Guertzenstein, S. P. Rigonatti

**Affiliations:** 1 Neurosurgery Division, Hospital das Clínicas da Faculdade de Medicina da Universidade de São Paulo; 2 Psychiatry, Instituto de Psiquiatria do Hospital das Clínicas da Faculdade de Medicina da Universidade de São Paulo, São Paulo, Brazil

## Abstract

**Introduction:**

Fibromyalgia (FM) is considered a chronic musculoskeletal pain condition of clinical complexity, likely resulting from dysfunction of central pain processing pathways. FM and depressive disorder share similar neurobiological mechanisms, particularly dysfunction of the hypothalamic-pituitary-adrenal (HPA) axis, which may be influenced by genetic inheritance or early developmental factors. The condition is characterized by widespread musculoskeletal pain, sleep disturbances, anxiety/depression, cognitive impairment, fatigue, and stiffness. Management of FM should primarily be non-pharmacological; however, pharmacological interventions may be indicated in generalized FM with increasing pain severity and additional symptoms. Patients experiencing severe pain and marked sleep disturbances may benefit from a multimodal therapeutic approach.

**Objectives:**

This prospective, randomized, single-blind, sham-controlled study aimed to evaluate the effects of electroconvulsive therapy (ECT) in drug-free patients with fibromyalgia.

**Methods:**

Patients were diagnosed according to ICD-11, DCR-10, and DSM-5-TR criteria. Clinical assessment included the Impact of Event Scale-Revised (IES-R, 22 items) and the Hamilton Depression Rating Scale (HAMD-17).

Functional impact was evaluated using the Fibromyalgia Impact Questionnaire (FIQ), Portuguese and Brazilian versions.

Assessments were performed at three time points: T0 (before initiation of ECT), T1 (after the 6th treatment session), and T2 (after the 12th treatment session).

Patients were randomly assigned to receive either active ECT (n = 162) or sham ECT (n = 163) in a simple randomization design.

Patients presenting with anxious symptoms (AD) following fibromyalgia were treated over a six-week period with a total of 12 sessions of transcranial direct current stimulation (tDCS). Each session lasted 30 minutes and was conducted twice weekly. Two bifrontal electrodes were placed on the scalp, delivering 2 mA with the anode positioned over the left dorsolateral prefrontal cortex (DLPFC) and the cathode over the right DLPFC.

**Results:**

Active ECT (A):

After the 12th treatment session (T2), 150 patients achieved complete remission of fibromyalgia (FM) and remained asymptomatic throughout the three-year follow-up. Additionally, 12 patients showed partial symptom remission after the 6th treatment (T1), which persisted through T2.

Sham ECT (S):

At T2, 155 patients showed no remission of FM. One patient experienced partial remission after T1 but relapsed after T2. Seven patients discontinued treatment.

**Conclusions:**

Electroconvulsive therapy (ECT) was effective in achieving remission of fibromyalgia symptoms in 150 patients, altering the clinical course regardless of whether patients presented with pre-FM depressive disorder, post-FM depressive symptoms (AD), pre-FM anxiety disorder, or post-FM anxiety symptoms (AD).

**Disclosure of Interest:**

None Declared

